# Anxious Profile Influences Behavioral and Immunohistological Findings in the Pilocarpine Model of Epilepsy

**DOI:** 10.3389/fphar.2021.640715

**Published:** 2021-04-30

**Authors:** Silvia Regina Bica Kohek, Maira Licia Foresti, Miriam Marcela Blanco, Clarissa Fantin Cavarsan, Clivandir Severino da Silva, Luiz E. Mello

**Affiliations:** ^1^ Physiology Department, Universidade Federal de São Paulo, São Paulo, Brazil; ^2^ Instituto D’ Or de Pesquisa e Ensino, Rio de Janeiro, Brazil; ^3^ Department of Biomedical and Pharmaceutical Sciences, College of Pharmacy, University of Rhode Island, Kingston, RI, United States; ^4^ George and Anne Ryan Institute for Neuroscience, University of Rhode Island, Kingston, RI, United States

**Keywords:** neuropeptide y, parvalbumin, seizure, temporal lobe epilepsy, epileptogenesis

## Abstract

Anxiety and epilepsy have a complex bidirectional relationship, where a depressive/anxious condition is a factor that can trigger seizures which in turn can aggravate the depressive/anxious condition. In addition, brain structures such as the hippocampus and amygdala might have a critical relevance in both epilepsy and anxiety. The aim of the present work was to investigate the influence of different anxious profiles to epileptogenesis. Initially, animals were screened through the elevated plus-maze anxiety test, and then seizure development was evaluated using the pilocarpine model of epilepsy. There were no differences in the susceptibility to status epilepticus, mortality rate or frequency of spontaneous recurrent seizures between animals characterized as anxious as compared to the non-anxious animals. Next, we evaluated immunohistological patterns related to seizures and anxiety in various related brain areas. Despite a decrease in the density of neuropeptide Y and parvalbumin expression in epileptic animals, those presenting greater neuropeptide Y immunoreactivity in various brain regions, also showed higher spontaneous recurrent seizures frequency. Differences on the anxious profile showed to interfere with some of these findings in some regions. In addition, animals that were injected with pilocarpine, but did not develop status epilepticus, had behavioral and neuroanatomical alterations as compared to control animals, indicating its importance as an additional tool for investigating the heterogeneity of the epileptogenic response after an initial insult. This study allowed to better understand the association between anxiety and temporal lobe epilepsy and might allow for therapeutic targets to be developed to minimize the negative impacts associated with it.

## Introduction

Epilepsy is a brain disorder that affects up to 1% of the world’s population (Moshé et al., 2015). Among all different epilepsy syndromes, the temporal lobe epilepsy (TLE) is the most common one (Löscher and Schmidt, 2002). Patients with TLE have a heterogeneous response to pharmacotherapy, which might be related to preexisting factors, and up to 50% of patients with TLE also have some associated psychiatric disorder, the most common being those related to mood, anxiety and psychosis (Torta and Keller, 1999; Thapar et al., 2009; Kandratavicius et al., 2012).

It has also been shown that some behavioral disturbances can also increase seizure susceptibility. To that end it has been reported that stress aggravates seizures in patients with epilepsy and may even stimulate acute convulsions in persons with no history of seizures (Christensen et al., 2007). Moreover, anxiety is associated with a variety of negative outcomes in persons with epilepsy, such as more severe epilepsy, debilitating seizures, and overall lower quality of life (Pham et al., 2017). In animals, it was observed that perinatal stress potentiates anxiety and the vulnerability to epileptogenesis (Salzberg et al., 2007; Hashemi et al., 2013; Rodriguez-Alvarez et al., 2015). Therefore, mechanisms that link the two pathologies have been the subject of investigation.

In common, psychiatric disorders and TLE present the involvement of similar brain structures, such as limbic circuits including amygdala and hippocampus, which are associated with neuroplastic changes such as neurodegeneration, synaptogenesis/sprouting and neurogenesis (Cendes et al., 1994; Pitkänen et al., 1998; Drevets, 2000; Satishchandra et al., 2003; Aroniadou-Anderjaska et al., 2008; Yilmazer-Hanke et al., 2016). Supporting this knowledge, direct stimulation of limbic structures, may trigger seizures, and these same limbic regions are also clearly involved in emotional behaviors (Racine, 1972; LeDoux, 1992; Helfer et al., 1996; Kalynchuk et al., 1998; Adamec and Young, 2000; Fanselow and Gale, 2003; Gröticke et al., 2007).

The major cause of hyperexcitability in TLE is the imbalance between inhibitory and excitatory synaptic transmission, which involves altered levels of the neurotransmitter’s glutamate and GABA, besides altered balance of additional neuromodulators also seems to influence the development of these pathologies. It has been described that in epileptic conditions, there is a robust overexpression of a tyrosine-rich polypeptide, neuropeptide Y (NPY) and NPY receptors, mainly in granular and pyramidal cells of the hippocampus, contributing to the tonic inhibition of glutamate release and, consequently, controlling the excitability that would spread to other brain structures (Xapelli et al., 2006). Similarly, increased parvalbumin (PV) immunostaining at several brain sites was related with seizure development in the lithium–pilocarpine model (Wolf et al., 2016). Additional studies also reported altered expression of NPY and PV in the context of epilepsy (Vezzani et al., 2002; Yekhlef et al., 2015) and behavioral changes associated with it, including anxiety (Frisch et al., 2009; Wu et al., 2011; O’Loughlin et al., 2014; Zou et al., 2016), corroborating findings linking these neuromodulators with both pathologies.

In this work we investigated the interrelationships between anxiety and epilepsy, more specifically the influence of previous anxiety levels on different aspects of epileptogenesis, such as neuronal loss and expression of selective neuromodulators. For this, animals were screened with regard to anxiety behavior in the elevated plus-maze test, and were subsequently submitted to the pilocarpine model of TLE. After the emergence of spontaneous seizure, the expression of NPY and PV was evaluated on different limbic structures associated with both epileptogenesis and anxiety. The results show that the anxious profile does not alter the spontaneous recurrent seizures development but influences the NPY and PV cell density in different brain regions after pilocarpine induced SE.

## Material and Methods

### Experimental Design

All experiments were approved by the Ethics Committee on Animal Research of UNIFESP (CEUA n: 0114/08). Adult male Wistar rats (180–240 g, 8–12 weeks age) were provided by the University Central Animal House (CEDEME) and maintained under controlled conditions (12/12 light/dark cycle, lights on 07:00 a.m.), with rat chow pellets (Nuvilab, Colombo, Brazil) and water available *ad libitum*.

**FIGURE 1:**
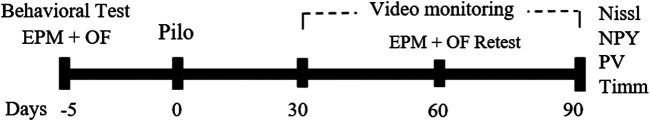
Schematic representation of the experimental timeline. Five days prior the pilocarpine-induced SE (day 0), animals were subjected to the behavior test. Thirty days after the SE, animals were video monitored. Sixty days after SE induction, a behavior retest was performed (day 60), and ninety days after SE, animals were perfused and their brains processed for Nissl and neo-Timm staining, and for immunohistochemistry against NPY and PV. EPM, elevated plus maze; OF, open field; NPY, neuropeptide Y; PV, parvalbumin.

Initially, animals were screened using the elevated plus maze (EPM) test and grouped in accordance with their anxiety level. After being behaviorally categorized, animals were injected with pilocarpine and were video monitored for SE and for ensuing spontaneous recurrent seizures (SRS). All behavioral tests and pilocarpine injections were performed during the morning period (8–12 h a.m.). Two months after pilocarpine injection, the behavioral condition of a subset of animals was reevaluated with the EPM. Histological processing was then performed, and the brain tissue was analyzed for different parameters 3 months after SE induction. Protocol details are presented in Figure 1.

### Groups Selection–Behavioral Tests

#### Elevated Plus Maze

The apparatus consisted of two opposite open arms (50 × 10 cm) and two opposite enclosed arms (50 × 10 × 40 cm), 50 cm above the floor. The junction area of the four arms (central platform) measured 10 × 10 cm. Each animal was placed on the center of the maze facing an open arm, and the time of permanence and the number of entries in the open and closed arms were analyzed for 5 min. Arm entry was defined as the entry of all four paws into an arm. With these parameters, it was possible to calculate the total number of entries, the percentage of entries and the time spent in the open arms in relation to both open and enclosed arms (Pellow and File, 1986). Ethanol 20% was used to clean the arena between each animal test.

Upon conclusion of the behavioral testing, the results were immediately calculated, and two animal groups were formed: Anxious (Anx) and Non-Anxious (Non-Anx). For this we used the ‘anxiety index’ (ITO), that expresses the total time spent in the open arms relative to the total time spent in both arms (open and closed). Animals with ITO ≤20% were classified as anxious (Anx) and animals with ITO ≥25% formed the non-anxious group (Non-Anx) (Harro et al., 1990; Ribeiro et al., 1999; Ho et al., 2002, 2005; Wilson et al., 2007). Animals presenting an intermediary ITO (20%<ITO<25%) were excluded from subsequent experiments.

#### Open Field

The apparatus consists of an arena of white wood (150 cm diameter) enclosed by walls and divided in 19 squares by black lines. Each animal was placed in the center of the arena and during 5 min the number of squares crossed (with four paws), the time spent in different areas of the field (periphery or center) and the total amount of rearing, freezing and grooming were counted (Walsh and Cummins, 1976). After completion of each session for a given animal, ethanol 20% was used to clean the arena. Locomotor activity through the open field (OF) test was assessed for potential interference in group selection and was then dismissed as a potential source of influence.

### Pilocarpine-Induced Seizures

Five days after behavioral testing, animals from both groups (Anx and Non-Anx) were injected with pilocarpine for SE induction, as described elsewhere (Mello and Covolan, 1996). Briefly, scopolamine methyl bromide (1 mg/kg, i.p., Sigma) was administered, 30 min prior to pilocarpine hydrochloride (320 mg/kg, i.p., Merck), to reduce peripheral effects. Some animals presented a generalized convulsive (stage 4 or 5) seizure that turned into continuous seizures in the form of limbic motor seizures with intense salivation, rearing, upper extremity clonus and falling, lasting up to 90–150 min, which characterized SE (Mello and Covolan, 1996). Thionembutal (25 mg/kg, i.p., Cristalia, Brazil) was administered 90 min after SE onset to suppress or attenuate SE. On the following 2–3 days after SE, animals were fed with a mixture of sweetened milk and Gatorade at least three times/day. During this 2–3 day period, fruit slices (bananas and oranges) were placed on the floor of the cage. Animals that received pilocarpine but did not progress into continuous limbic seizure activity (NoSE), also received thionembutal. The control group was injected with an equivalent amount of saline followed by injection of sodium thionembutal 90 min later.

### Spontaneous Recurrent Seizures Development

Beginning one month after pilocarpine induced SE, animals were video monitored for spontaneous recurrent seizures (SRS). The behavioral recordings were sampled 12 h/day, during the daytime (7:00 am–7:00 pm), in which seizures are most likely (Arida et al., 1999), for 5 days/week, over a period of 2 months. Spontaneous seizures ranging from stages 3–5 (according to Racine) were considered for statistical analysis. The SRS frequency was calculated as the total number of SRS relative to the total amount of recorded hours (∼720 h/animal).

### Behavioral Tests at the Chronic Phase (Retest Session)

Sixty days after pilocarpine injection, a subset of rats was retested for both EPM and OF tests, to verify the seizure impact on animal´s anxiety levels. Behavioral tests were performed as previously described and results of the assessments before and after the pilocarpine injection were compared within groups.

### Histological Analysis

Three months after SE induction, animals received a lethal dose of thionembutal (50 mg/kg, i.p.) and were transcardially perfused with 25 ml Millonig’s buffer 0.12 M followed by 50 ml Na_2_S 0.1% and 100 ml glutaraldehyde 3%. After removal from the skull, the brains were post fixed at 4°C and cryoprotected in a 30% sucrose solution for 48 h. Coronal sections of 30 μm were cut on a cryostat and stored in an anti-freezing solution until the staining procedures.

#### Nissl Staining (Cresyl-Violet)

The neuronal damage in the hilus of the hippocampus was evaluated using Nissl-stained sections (cresyl-violet 0.4%). The identified nuclei of neuronal cells were counted in three consecutive slices at mid level of the hilar region of the dorsal hippocampus in both hemispheres, corresponding to plate 30 of the rat brain atlas (Swanson, 2004), at ×40 objective with ×10 digital zoom (×400 magnification) over a microscope grid (Olympus B×50) corresponding to a selected area of 1000 μm^2^. The results attributed to each animal represent the mean ± SEM of both hemispheres and are presented as cells/mm^2^.

#### Immunohistochemistry for Neuropeptide Y and Parvalbumin

Immunohistological assessment was also made for NPY, as described by Schwarzer et al,. 1996 and for parvalbumin, as described by Yew et al,. 1990. After suppressing the endogenous peroxidase activity, the free-floating sections were incubated with the primary antibody (rabbit anti-NPY, 1:3,000, Sigma N9528, for 48 h; mouse anti-PARV, 1:1,000, Sigma P3088, for 24 h) in a solution containing blocking buffer (3% bovine fetal serum, 0.2% Triton X-100 in PB). Immunostaining was visualized using a biotinylated secondary antibody for 2 h (1:200, Vector), the avidin-biotin complex and revealed with diaminobenzidine tetrahydrochloride (DAB, 1 mg/1 ml). Cell countings were performed on three consecutive slices per animal using a ×40 objective with ×10 digital zoom (×400 magnification, Olympus B×50). On each slice, a microscope grid was placed over three different sample areas (1000 μm^2^) per region, which included the pyramidal layer of CA1/CA3, granular layer of dentate gyrus, hilar region, basolateral and basomedial amygdala, entorhinal and piriform cortex, from rostral to dorsal levels (between Plates 26 and 42; Swanson, 2004). The result attributed to each animal was an average value, from both hemispheres, and described as the total number of neurons per mm^2^.

#### Neo-Timm Staining

Neo-Timm staining was used to evaluate mossy fiber sprouting. After the monitoring period, animals were submitted to Neo-Timm staining as previously described (Queiroz e Mello, 2006). The slices were immersed in a 360 ml solution containing 240 ml of arabic gum, 10.25 g citric acid, 9.45 g sodium citrate, 3.73 g hydroquinone and 510 mg of silver nitrate for approximately 45 min, at 21°C. Visual inspection of slices was used to control the reaction time. To eliminate potential bias associated with different reaction times, slices from control and experimental groups were processed in the same batch. The sections used for the Neo-Timm assessment were immediately adjacent to the ones used for the cell counts. An optical density analysis was performed with a computer-assisted system to measure the intensity of the mossy fiber sprouting. Initially, the brain section was magnified by means of a microscope (Olympus B×50) coupled to a video camera (Sony CCD-Iris). The image was then digitized to the NIH Image 1.62 software. The software analyzed the gray level of every pixel of the digitized image, which could vary from 1 (white) to 256 (black). The evaluations were performed in the molecular layer of the dentate gyrus. Three adjacent sections per animal were evaluated in the dorsal (plate 30) and ventral (plate 40) hippocampus (Swanson, 2004).

#### Statistical Analysis

The number of animals (n) used for the different tissue analysis is summarized on Table 1. For other analysis, the *n* is described where appropriate. Data normality was verified with the Shapiro-Wilk test and homogeneity by the Levene’s test. SE incidence and mortality rate were compared with Fisher's exact test. Cell counting and densitometry analyses were compared by the Two-way analysis of variance (ANOVA) followed by Bonferroni post hoc test and were expressed as mean ± SEM, unless otherwise stated. For comparisons between behavioral tests before and after pilocarpine injection it was used paired *t*-test. We investigated the association between the different immunohistochemical data and SRS occurrence by means of the Pearson Correlation test. Statistical significance was accepted at *p* < 0.05.

**TABLE 1:** Number of animals (*n*) per group used for different tissue analysis.

Structure	Group	NPY			PV			Nissl		
		Control	NoSE	SE	Control	NoSE	SE	Control	NoSE	SE
DG	Anx	3	15	29	4	5	15			
	Non-Anx	3	10	13	5	5	11			
Hilus	Anx	3	15	29	4	5	15	4	13	23
	Non-Anx	3	10	13	5	5	11	5	7	13
CA3	Anx	3	15	29	4	5	15			
	Non-Anx	3	10	13	5	5	11			
CA1	Anx	3	15	29	4	5	15			
	Non-Anx	3	10	13	5	5	11			
BL	Anx	4	5	15	4	5	15			
	Non-Anx	5	5	11	5	5	11			
BM	Anx	4	5	15	4	5	15			
	Non-Anx	5	5	11	5	5	11			
PIR	Anx	4	5	15	4	4	15			
	Non-Anx	4	5	11	5	5	11			
ENT	Anx	3	5	14	4	5	15			
	Non-Anx	3	4	9	5	4	11			

## Results

The EPM test was initially used to screen and allocate the animals according to the anxiety level. Of the total 146 animals tested, 83 were classified as anxious (Anx, ITO≤20%) and 44 were classified as non-anxious (Non-Anx, ITO≥25%). Additional 19 animals presented an intermediary ITO (20%<ITO<25%) and were excluded from subsequent experiments. In addition to the increased time spent in the open arms (10.4 ± 0.9 and 33.2 ± 1.7, respectively; Student *t* test, *p* < 0.01), Anxious animals also presented increased number of entries in the open arms as compared to Non-Anxious animals, therefore confirming the difference in the anxiety parameters between the two groups.

Five days after the EPM test, nine animals were allocated as controls (Anx Control *n* = 4, Non-Anx Control *n* = 5) and 118 animals were injected with pilocarpine. Of the animals injected with pilocarpine in the Anx group (*n* = 79), 44% did not present SE (Anx NoSE) with the remaining 56% developing SE (Anx SE). Twenty one of the 44 Anx SE animals (48%) died and 87% of the 23 surviving animals developed SRS during the follow up period. For animals injected with pilocarpine in the Non-Anx group (*n* = 39), 49% did not present SE (Non-Anx NoSE) and 51% developed SE (Non-Anx SE). Seven of the 20 Non-Anx SE animals died (35%) and 92% of the 13 surviving animals developed SRS during the follow up period. Neither SE incidence, nor SRS development were influenced by the animals´ baseline anxiety level as measured by the EPM test (Fisher's exact test, *p* > 0.05). Similarly, the SRS frequency and duration also did not vary between the different groups (Student *t* test, *p* > 0.05; data not shown).

Sixty days after pilocarpine injection, a subset of animals was again exposed to the EPM and to OF tests. Non-Anx animals that did not develop SE (Non-Anx/NoSE) presented a profile similar to that of anxious animals at the re-test, as indicated by reduced parameters of ITO and INO as compared with the levels evaluated before pilocarpine injection (Paired *t*-test, *p* < 0.05, Table 2). This difference was not observed for other groups (Table 2).

**TABLE 2:** Behavioral tests before and after (60 days) pilocarpine injection.

Behavoral Test	Parameter	Anx				Non-Anx			
		NoSE (*n*=12)		SE (*n*=5)		NoSE (*n*=7)		SE (n=4)	
		BEFORE	AFTER	BEFORE	AFTER	BEFORE	AFTER	BEFORE	AFTER
EPM	ITO % INO %	12±1.5	14±3.7	14±2.7	18±13.9	27±3.8	8±3.8*	26±3.7	70±10
		32±1.9	29±5.4	33±6.8	25±12.6	40±2.2	17±5.6*	42±2.4	70±11.7
Open Field	Locomotion (frequency)	68±5.8	49±5.1*	73±8.6	50±37.5	80±5.9	35±9*	78±10.9	51±49
	Rearing (frequency)	19±1.6	18±3	25±2.5	0.7±0.7*	23±2.9	11±2.2*	34±4.0	6±6.0*
	Freezing (frequency)	2±0.7	0.5±0.3*	2±0.8	0.2±0.2	0.4±0.4	0.7±0.5	2±1.0	1±0.7
	Grooming (s)	17±4.3	5±1.8*	12±1.4	2.2±2.2*	7±2.3	17±9.7	16±4.6	2.5±2.5
	Time in center (s)	19±14.6	26±7.3	13±4.3	131±85.3	17±2.2	23±7	15±6.4	156±144*

EPM, elevated plus maze. ITO, relation between the time spent in the open arms and the time spent in both arms; INO, relation between the number of entries in the open arms and the total number of entries. Locomotion, frequency that animals crossed the different delimited fields in the open field arena.Values: mean ± SEM, *p ≤ 0.05 before vs after (Paired *t*-test).

Rats that developed SE regardless of anxiety levels (both Non-Anx and Anx) presented less rearing frequency in the OF test as compared to the levels evaluated before pilocarpine injection (Paired *t*-test, *p* < 0.05, Table 2). Other behavioral parameters were also found to be reduced after pilocarpine in different animal groups (Table 2) which may be indicative of the animals' habituation to the test or alternatively emotional dullness resulting from pilocarpine injection (Table 2).

**FIGURE 2:**
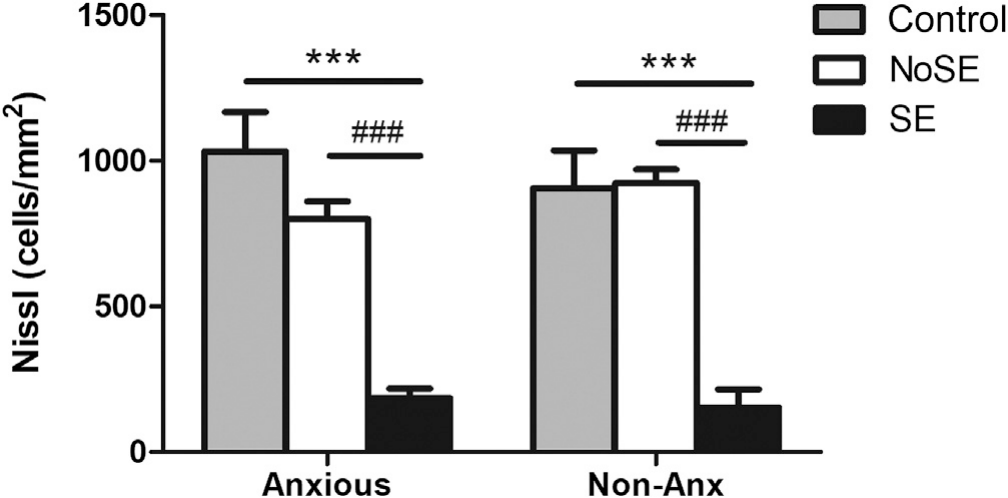
Density of neuronal cells in the hilus 90 days after pilocarpine injection. SE animals presented decreased neuronal density as compared to controls and NoSE animals in both Anx and Non-Anx groups. Control animals and NoSE animals, in both Anx and Non-Anx groups, showed similar amounts of cells. Values: mean ± SEM. Two-Way ANOVA, Bonferroni post hoc test, Seizure *p* < 0.05 *vs Controls, #vs NoSE; Anxiety *p*>0.05; Interaction *p*>0.05.

**FIGURE 3:**
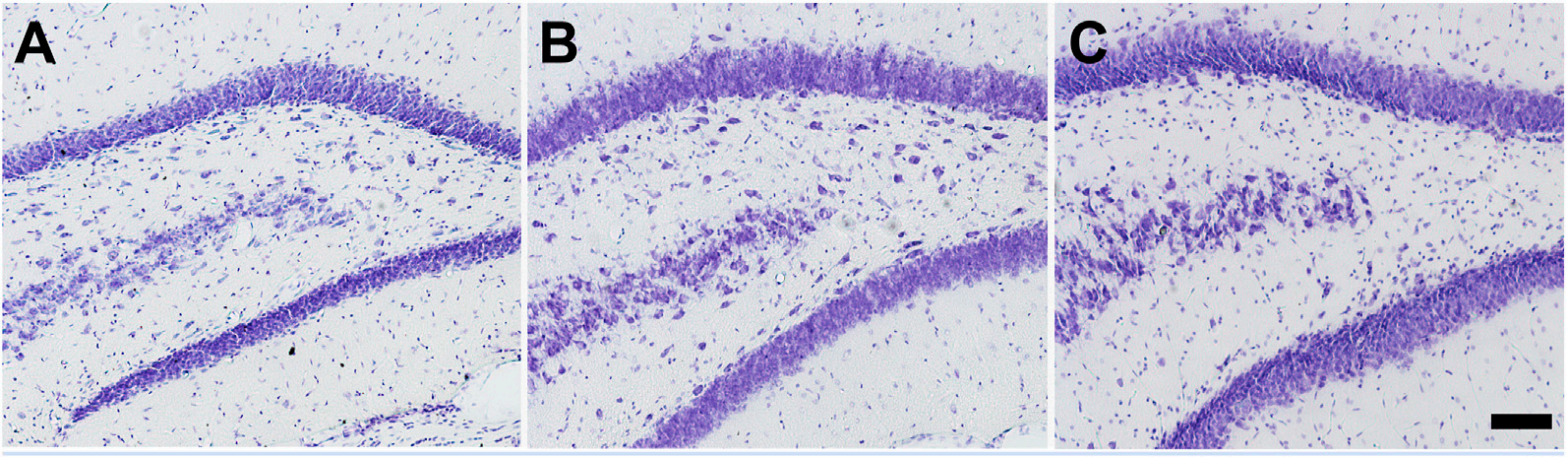
Photomicrographs of Nissl staining in the hilus of the hippocampus 90 days after pilocarpine injection. **(A)** Control **(B)** NoSE **(C)** SE animal. Note intense neuronal loss in SE group as compared to controls and NoSE groups. Scale bar: 100 μm.

The hilar neuronal density also did not differ as a function of the animals´ anxiety condition or of the pilocarpine injection (when not inducing SE). However, animals that presented SE in both Anx and Non-Anx groups exhibited neuronal loss in the hilus 3 months after pilocarpine injection (TwoWay ANOVA, Bonferroni post hoc test, *p* < 0.05, Figures 2, 3).

**FIGURE 4:**
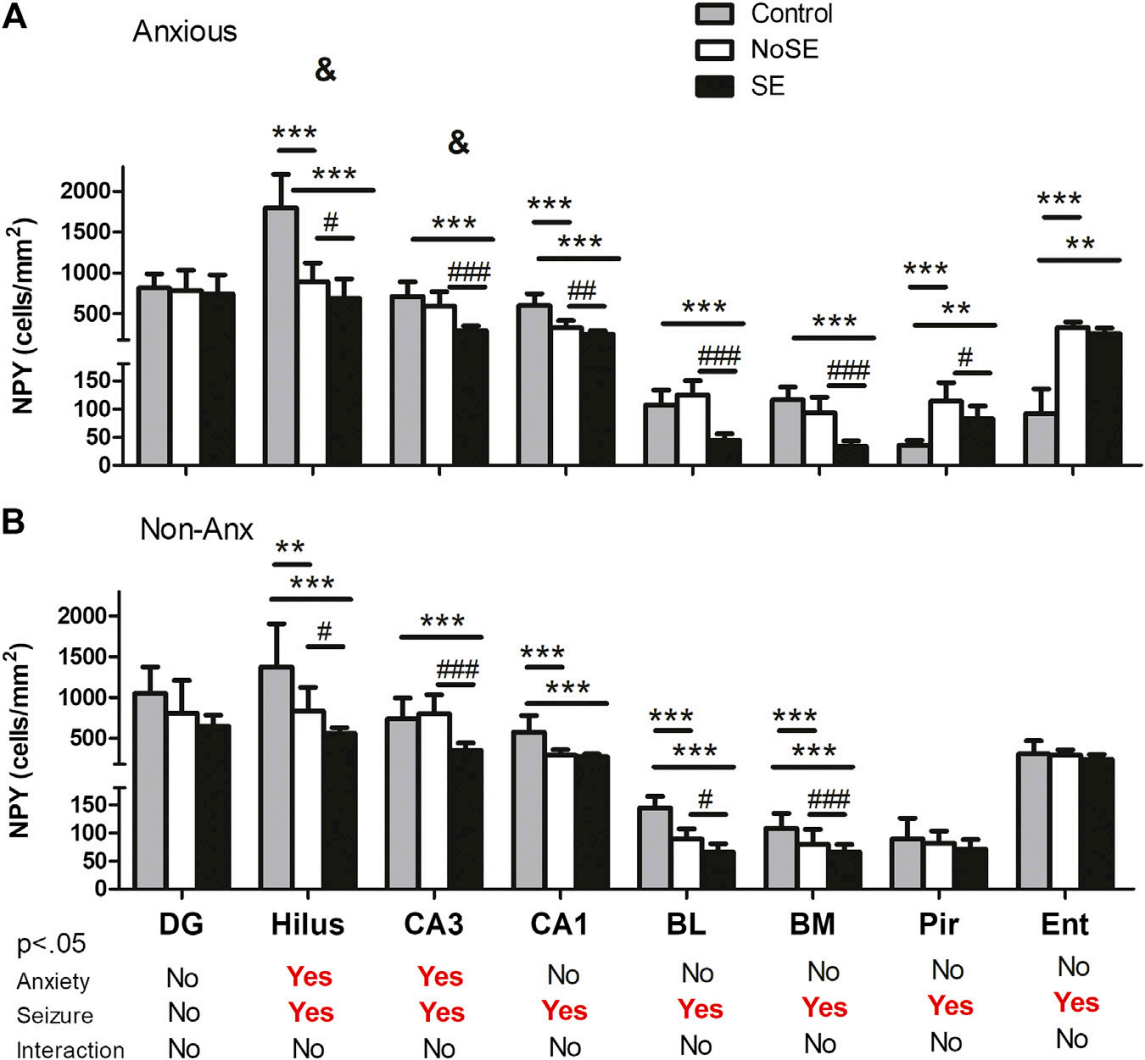
Density of NPY + cells in several regions 90 days after pilocarpine injection. **(A)** Anxious group. **(B)** Non-Anxious group. The NPY + cell density in the hilus and CA3 hippocampal regions was influenced by anxiety (^&^A vs B). Most differences occurred within groups, where pilocarpine injection and SE development influenced the NPY + cell density as compared to controls (except for the dentate gyrus, and the piriform and entorhinal cortices in Non-Anx animals, A and B). Values: mean ± SEM. Two-Way ANOVA, Bonferroni post hoc test, ^&^ *p* < 0.05 Anxious vs Non-Anxious; SE group vs Controls (**p < 0.01, ***p < 0.001) and SE group vs NoSE (^#^ *p* < 0.05, ##*p* < 0.01, ###*p* < 0.001). NPY, neuropeptide Y; DG, dentate gyrus; BL, basolateral amygdala; BM, basomedial amygdala; Pir, piriform; Ent, entorhinal cortex.

**FIGURE 5:**
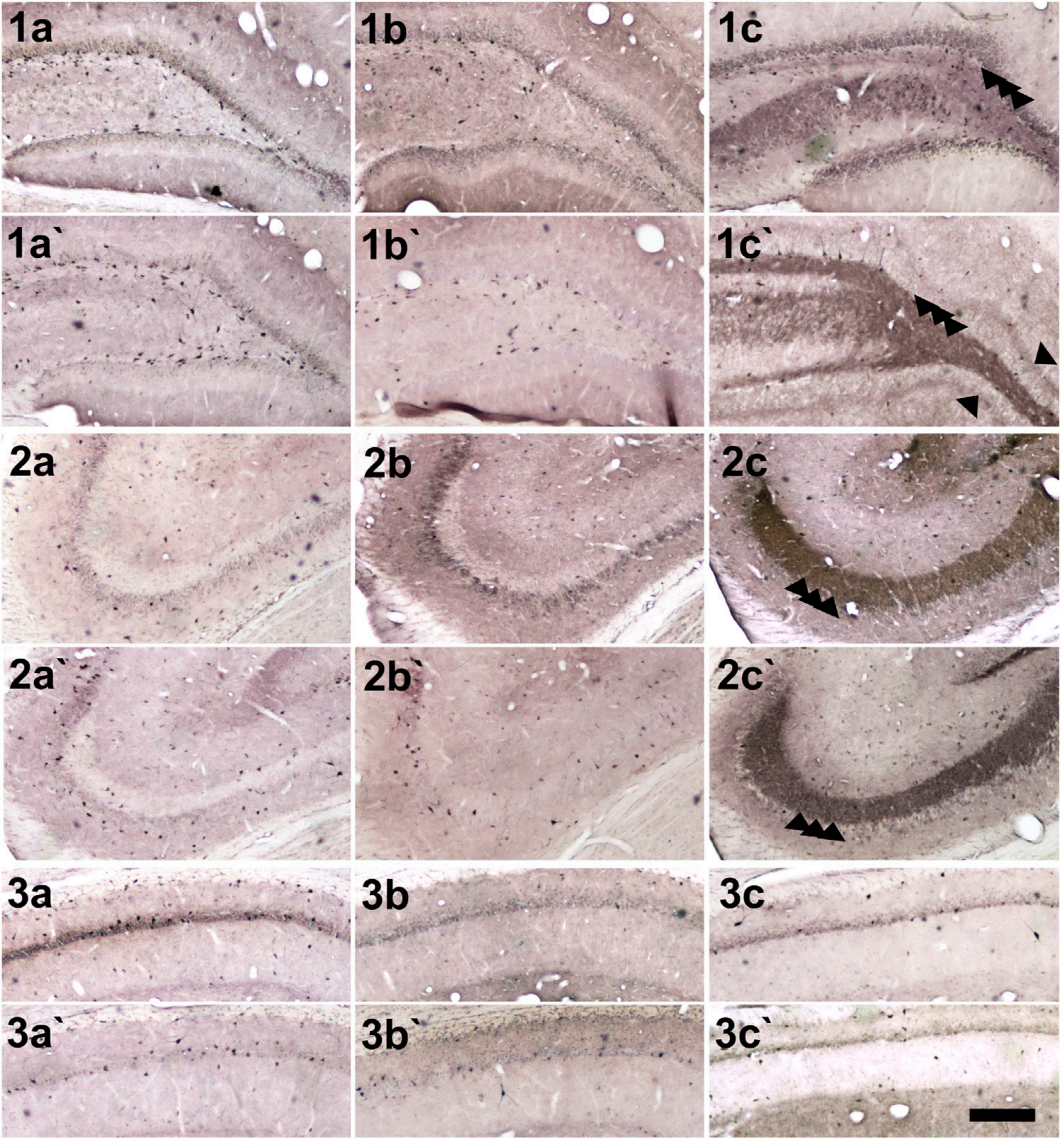
Photomicrographs of NPY + cells in hippocampal regions. (1) Dentate gyrus and hilus, (2) CA3 and (3) CA1. (a, b and c) Anx (a`, b` and c`) Non-Anx (a, a`) Controls (b, b`) NoSE (c, c`) SE. Note intense (darker) and widespread staining in regions which coincide with mossy fiber sprouting in the dentate gyrus, hilus and CA3, exclusively in SE animals (arrow heads). Scale bar: 250 μm.

**FIGURE 6:**
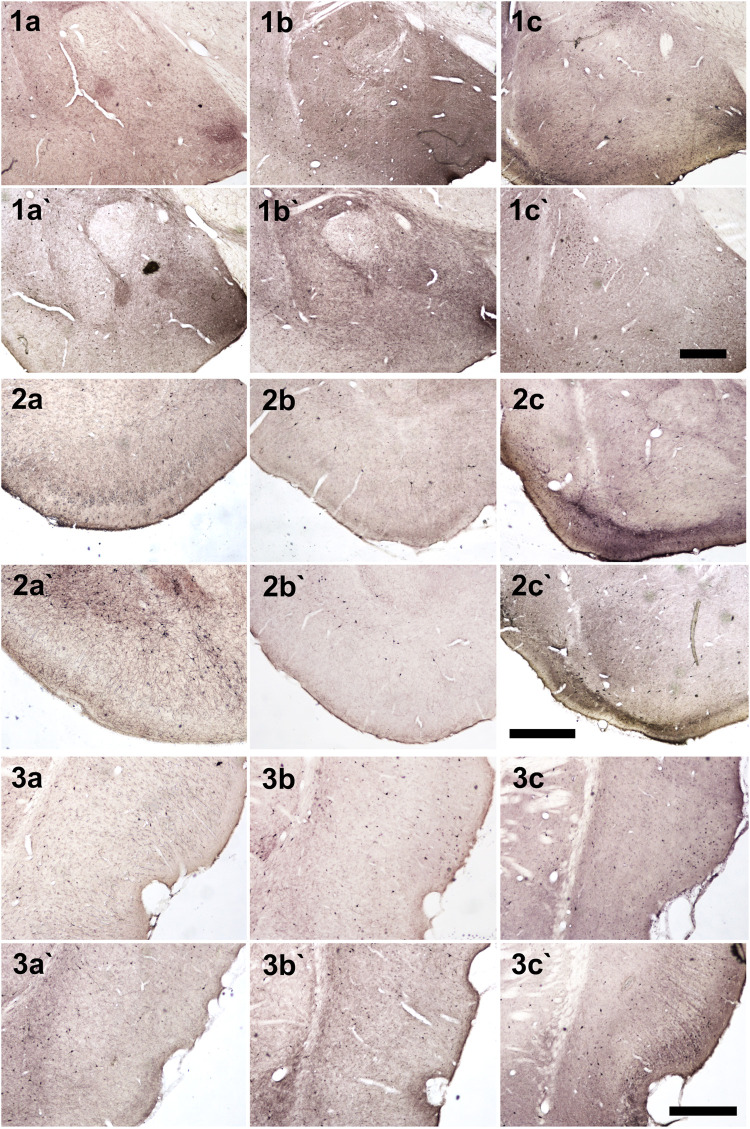
Photomicrographs of NPY + cells in different brain regions. (1) Amygdala, (2) Piriform cortex, (3) Entorhinal cortex (a, b and c) Anx (a`, b` and c`) Non-Anx (a, a`) Controls (b, b`) NoSE (c, c`) SE. Scale bars: 500 μm.

**FIGURE 7:**
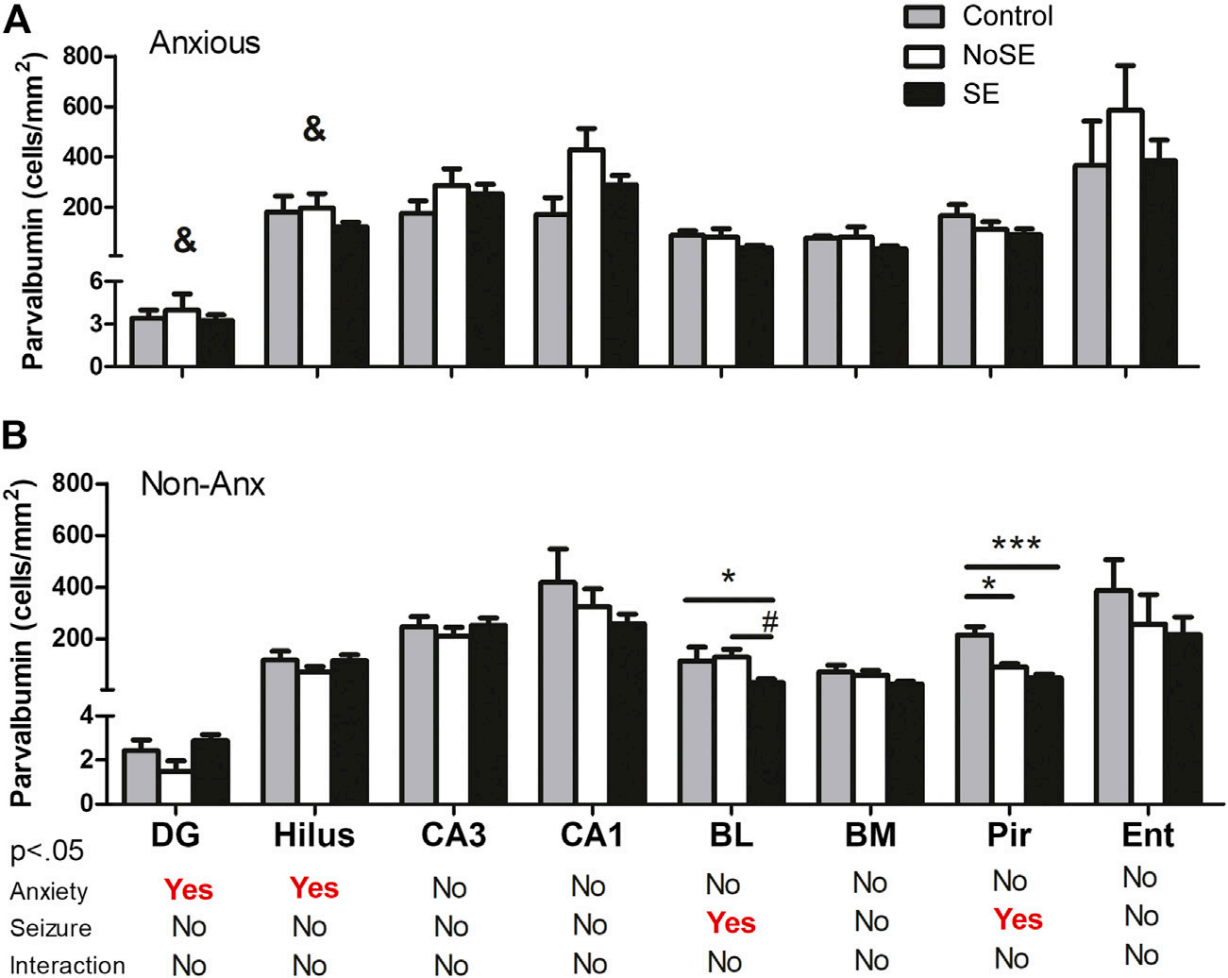
Density of PV in several regions 90 days after pilocarpine injection. **(A)** Anxious group **(B)** Non-Anxious group. The PV density in the dentate gyrus and hilus was influenced by the anxiety (&A vs B). Also, pilocarpine injection and SE development influenced PV density in the basolateral amygdala and piriform cortex, but only in Non-Anx group **(B)**. Values: mean ± SEM. Two-Way ANOVA, Bonferroni post hoc test, *p* < 0 .05 &Anxious vs Non-Anxious; SE group vs Controls (*p < 0.05; ***p < 0.001) and SE group vs NoSE (^#^ *p* < 0.05); Interaction *p* > 0.05. Parvalbumin, PV; DG, dentate gyrus; BL, basolateral amygdala; BM, basomedial amygdala; Pir, piriform; Ent, entorhinal cortex.

The anxiety condition influenced the density of NPY + cells in the hippocampus, more specifically at the hilus and CA3 regions (Two-way ANOVA, Bonferroni post-hoc test, behavior *p* < 0.05, Figure 4). Except for the dentate gyrus, in all other analyzed regions the pilocarpine injection showed to interfere with NPY + cell density, both in SE and NoSE groups (Figures 5, 6). Interestingly, this difference was observed in both Non-Anx and Anx groups in the hippocampus, while in the piriform and entorhinal cortices NoSE animals presented less NPY + cells as compared to control animals only in the Anx groups, and only in the Non-Anx group in the amygdala (Two-way ANOVA, Bonferroni post-hoc test, seizures *p* < 0.05, Figure 4). It is important to highlight the evident more intense (darker) and widespread staining in regions which usually coincide with mossy fiber sprouting in the dentate gyrus, hilus and CA3 that occurred only in SE animals, both in Anx and Non-Anx groups (Figure 5; compare Figure 5 (1c and 1c'), with Figure 7C). There was a positive correlation between the NPY density and the frequency of SRS in the CA1 region, piriform and entorhinal cortices in Anx groups, and in the hilus of Non-Anx animals (Pearson Correlation, r > 0.04, *p* < 0.05, Table 3). This correlation was not significant for other brain structures (Table 3).

**TABLE 3:** Correlation between NPY and PV density and SRS frequency.

	Group	DG	Hilus	CA3	CA1	BL	BM	Piriform	Entorhinal
NPY	Anx	0.02	0.22	0.35	0.48*	0.34	0.14	0.58*	0.60*
	Non-Anx	0.49	0.59*	0.12	0.44	−0.04	0.42	−0.03	−0.21
PV	Anx	0.39	0.21	−0.28	−0.08	−0.21	0.39	0.21	−0.28
	Non-Anx	0.63*	0.06	0.03	−0.10	−0.22	−0.25	−0.25	−0.19

Values: Pearson r, *p < 0.05. Anx (*n* = 14–23) Non-Anx (*n* = 9–13).

**FIGURE 8:**
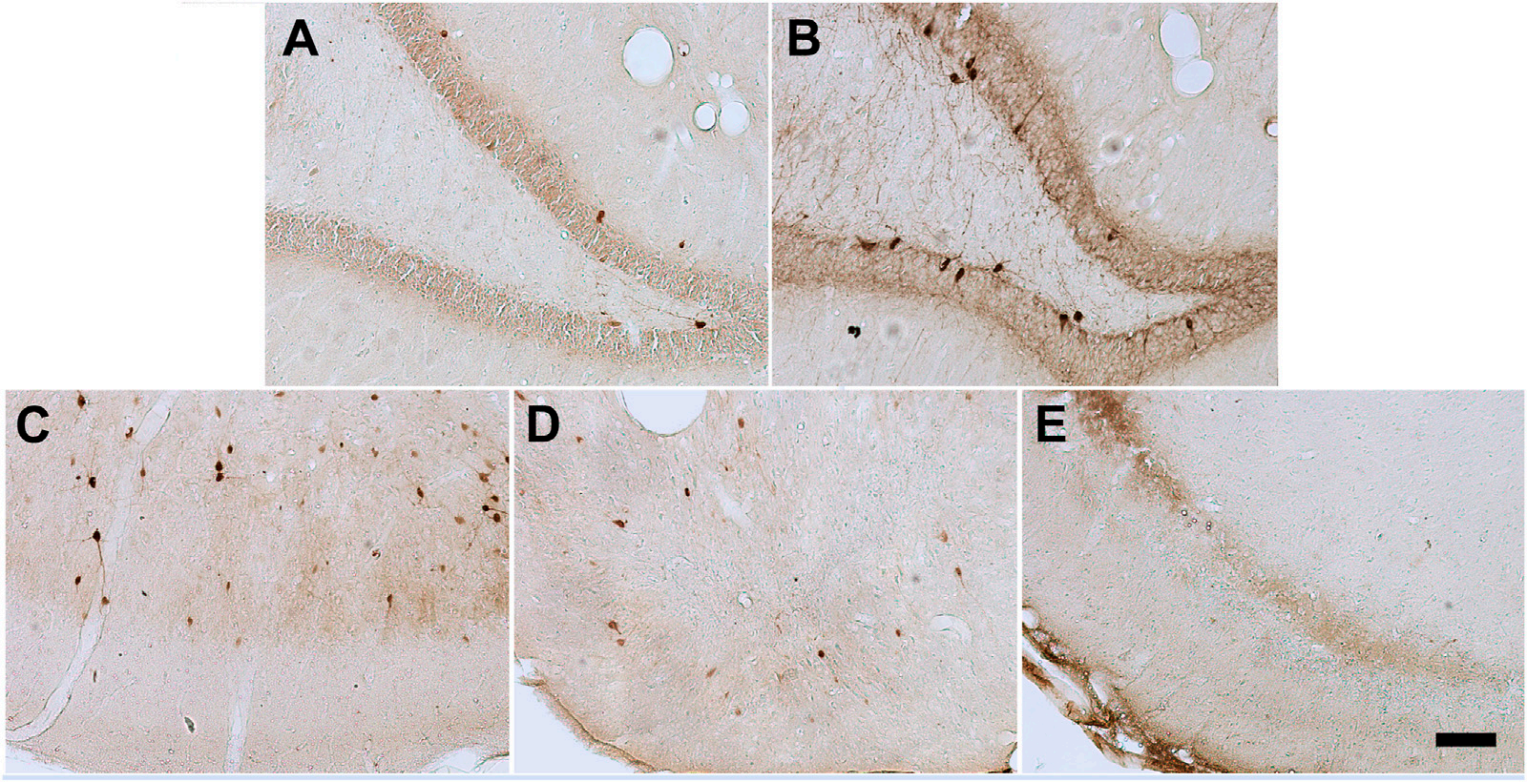
Photomicrographs of PV + cells in the different brain regions. Hippocampal dentate gyrus and hilus in Non-Anxious **(A)** and Anxious **(B)** animals. Piriform cortex in Control **(C)**, NoSE **(D)** and SE **(E)** animals. Note increased number of PV + cells in the animal of the Anx group as compared to Non-Anx group and decreased PV density in NoSE and SE animals as compared to Controls. Scale bar: 100 µm.

**FIGURE 9:**
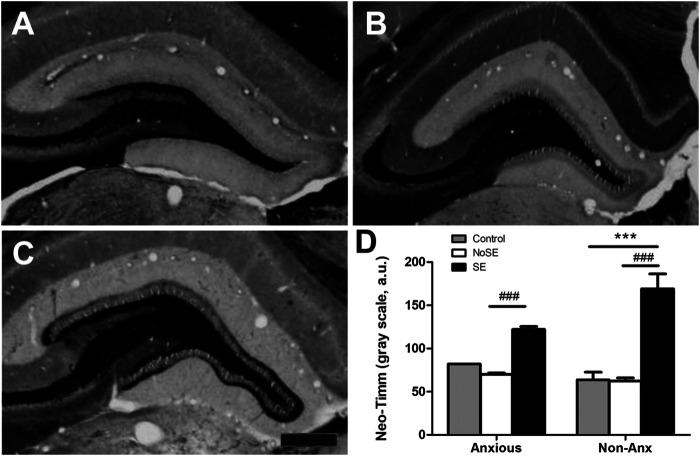
Photomicrographs of Neo-Timm staining in the hippocampal dentate gyrus. **(A)** Control **(B)** NoSE **(C)** SE animal. Note slightly increase of Timm staining in NoSE animals and evident darker staining, especially at the inner molecular layer of the dentate gyrus in SE animals. **(D)** Densitometry analysis of the inner molecular layer confirmed increased Neo-Timm intensity in SE animals and no influence of the anxiety level on mossy fiber sprouting. Values: mean ± SEM. Two-Way ANOVA, Bonferroni post hoc test, *p* < 0.05 SE groups *vs Controls, ^#^vs NoSE. Scale bar: 50 μm.

The parvalbumin density varied in some of the analyzed structures, sometimes being influenced by the anxious condition and other times by the SE development. In both the dentate gyrus and hilus the behavioral condition showed to influence the results, since the parvalbumin density varied among Anx and Non-Anx groups (Two-way ANOVA, Bonferroni post-hoc test, *p* < 0.05; Figures 8, 9) and were independent of the SE development. In the piriform cortex, Non-Anx/NoSE group presented decreased parvalbumin density as compared to controls (Figure 8). Meanwhile, in both the piriform cortex and basolateral amygdala, the SE development significantly decreased the parvalbumin density in the Non-Anx groups (Figure 8). There was a positive correlation between the PV density and the frequency of SRS in the dentate gyrus of Non-Anx animals (*n* = 11, Pearson Correlation, *r* = 0.6, *p* < 0.05) which was not observed for other regions and groups (Table 3).

Densitometry analysis of Neo-Timm staining, showed increased hippocampal mossy fiber sprouting in the dentate gyrus of SE animals, as compared to controls, 90 days after pilocarpine injection (TwoWay ANOVA, Bonferroni post hoc test, *p* < 0.05, Figure 7). The anxiety condition did not influence this measurement (*p* > 0.05).

## Discussion

The present study investigated the association between anxiety and the development of epilepsy in rats. Our results indicate that animals with baseline anxious profiles (Anx), as assessed by the EPM test, did not exhibit greater susceptibility to the SE incidence, nor higher mortality rates and neither altered the development of SRS when submitted to the pilocarpine model of epilepsy. On the other hand, the anxious profile was shown to influence the NPY and PV cell density in specific brain regions, specially related to the animal’s epileptic condition. In addition, they also showed to influence the correlation between the expression of these neuromodulators and the frequency of SRS in epileptic animals.

The relationship between anxiety and epilepsy has been the subject of numerous studies (Vazquez and Devinsky, 2003; Kanner 2005; Pham et al., 2017). It is known that psychiatric disorders and epilepsy have a complex bidirectional relationship, where a depressive/anxious condition is a psychic factor that triggers seizures (Kanner, 2005). And, on the other hand, seizures would accentuate the depressive/anxious situation, thus creating a vicious circle. Of relevance, the same structures of the temporal lobe are related to both pathologies. These include the hippocampal formation, piriform and rhinal cortices, and also amygdala, which were all investigated in the present study.

In this sense, a natural consequence of SE, especially considering the classical pilocarpine protocol used in the present study, is the intense damage to various brain structures, mainly the limbic system. In addition, mossy fibers create new recurrent excitatory circuits by projecting back to the molecular layer of the dentate gyrus, forming a unique aberrant network in the epileptic brain (Dudek et al., 2006). In the present study, epileptic animals presented both classical findings, mossy fiber sprouting and limbic neuronal death, which were not influenced by the anxious profile. Nevertheless, we can not exclude that alterations also occurred on extra-temporal lobe regions naturally connected to the investigated structures, such as thalamic nuclei, basal ganglia, brainstem and others, which are also known to influence epilepsy (Bertram, 2014; Vuong and Devergnas, 2018; Bröer 2020).

Interestingly, seizures increase NPY levels in the hippocampus, both in inhibitory interneurons and in excitatory granular cells and mossy fibers, which do not normally contain the peptide (Vezzani et al., 1999). In the present study, SE animals presenting higher SRS frequency also presented higher NPY cell density in the hippocampus, and also in the piriform and entorhinal cortices. Moreover, epileptic animals presented intense NPY staining at the recurrent mossy fiber pathway, corroborating previous findings on this topic (Nadler et al., 2007). This overexpression of NPY in epileptic animals may represent an endogenous “dampening” mechanism, which would limit the release of excitatory neurotransmitters during hyperexcitability conditions, since NPY has been shown to block events related to seizures in hippocampal slices stimulated at high frequency (Colmers et al., 1991; Schwarzer et al., 1995) and therefore acting as an endogenous anticonvulsant. Accordingly, it is suggested that NPY may inhibit seizures via down-regulation of the functional expression of N-methyl-d-aspartate receptors (Dong et al., 2013).

Even so, as a natural consequence of the neuronal loss in the epileptic brain, there is also loss of neurons which express different neurotransmitters and neuropeptides, including NPY. Here, despite a sufficient number of remaining cells expressing NPY, allowing positive correlation with SRS frequency, there was a decreased density of NPY expressing cells, both in the hippocampus and in the amygdala, which were mostly related to the epileptic condition. Although there was no direct characterization of which cell types were expressing NPY in the present study, the decreased density of NPY is likely due to the neuronal loss in epileptic animals (Lurton and Cavalheiro, 1997; Vezzani et al., 1999). This corroborates another study, that described decreased hippocampal and amygdalar Y1 receptor expression, associated with neuronal loss on these same regions, and suggested it could negatively impact anxiety levels in the intrahippocampal kainate epilepsy model (O’Loughlin et al., 2014).

Interestingly, the expression of neuromodulators also differed among Anx and Non-Anx animals in specific hippocampal regions, namely in the hilus and CA3 regions for the NPY expression and in the dentate gyrus and hilus for the PV expression. In addition, the positive correlation between NPY density and SRS frequency in SE animals, was mainly observed in Anx animals (except in the hilus of Non-Anx animals that also showed this correlation). Together these data indicate that the basal anxious condition has an important influence on neuromodulators expression. It has been shown that NPY gene delivery to the thalamus or somatosensory cortex, produced sustained anti-epileptic effects in genetic generalized epilepsy model with absence seizures, by increasing Y2 receptor expression, while no effect was observed on anxiety behavior (Powell et al., 2018). Although we did not investigate NPY receptors, in support of our findings, there is evidence suggesting that changes in hippocampal receptors and/or neurotransmitters are also possible causes of anxiety (Klitgaard et al., 2002).

We now discuss whether PV + neurons would play some role in inhibiting the activity of pyramidal and granular hippocampal cells. It is known that chandelier cells, specialized GABAergic interneurons subtype that selectively innervate pyramidal neurons, constitute one of the main neuronal populations responsible for hippocampal neuronal inhibition (Somogyi et al., 1985; Soriano et al., 1993; Buhl et al., 1994). These cells, in addition to expressing GABA, also co-express PV and calbindin (DeFelipe, 1997). A dysfunction of chandelier cells can lead to excessive and synchronous discharges from large neuronal populations, resulting on epileptic seizures (Engel, 1996b; Prince, 1999). In fact, our data showed a decrease in the density of PV + cells in the BL amygdala and in the piriform cortex of SE animals as compared to control animals. These changes on PV-containing GABAergic neurons in limbic regions were also found by other authors (Fernandes et al., 1999; Covolan and Mello, 2000; André et al., 2001; Gorter et al., 2001). In these studies, it was observed loss of interneurons, especially those that expressed PV and calbindin, in the CA1 (Covolan and Mello, 2000; André et al., 2001) and CA3 stratum oriens (Fernandes et 1999) and in the hilar region (Gorter et al., 2001). It is known that a dysfunction in the activity of several neurotransmitters and neuropeptides can act as a facilitator for epileptic seizures and psychiatric diseases, as already discussed, and also corroborate our data, in which epileptic animals showed less density of cells expressing PV and NPY.

As occurred in the NPY analysis, SE animals presenting higher PV density in the dentate gyrus, also presented higher SRS frequency which supports the hypothesis of this neuromodulator serving to compensate and acting as an endogenous anticonvulsant. Corroborating our data, both neuronal loss and increased parvalbumin immunostaining at several sites correlated with spontaneous seizure frequency in the lithium–pilocarpine model (Wolf et al., 2016). Importantly, the decrease in the PV cell density in SE animals was only verified in the Non-Anx group. Moreover, the basal hippocampal PV density, specifically in the dentate gyrus and hilus, was higher in Anx animals as compared with Non-Anx animals. Again, the anxious profile seems to be an important factor to be considered in the analyses of the PV expression in the seizure-anxiety context.

Our evidence and various reports suggest that the abnormal functioning of GABAA receptors is of great importance to the pathophysiology of epilepsy and anxiety (Chapouthier and Venault, 2001; Lydiard, 2003). Indeed, there is an important participation of glutamate and GABA systems in many brain functions, and well characterized imbalances of those systems in epilepsy. This hypothesis is also supported by the fact that some substances, including both the GABAergic antiepileptic drugs, gabapentin, vigabatrin, tiagabine, valproate and pregabalin, as for AMPA antagonists, barbiturates, benzodiazepines and neuroactive steroids have both antiepileptic and anxiolytic properties (Pande et al., 2000; Beyenburg et al., 2001; Ashton and Young, 2003; Blanco et al., 2003; Carta et al., 2003; Kinrys et al., 2003; Lauria-Horner and Pohl, 2003; Rosenthal, 2003; Bektas et al., 2020). Our choice to investigate other molecules, such as NPY and PV, provides additional data which might be relevant for potential therapeutic targets in such conditions.

By not specifying exactly which cell types are expressing PV and NPY, and which receptors types are directly involved with the observed behavioral outcomes, constitutes a limitation of the present study. As already mentioned, even though we did investigate some of the main recognized structures related to temporal lobe epilepsy, alterations in extra-temporal structures could also help to explain some of the observed results. Thus, while our data suggest that neurotransmitter systems in different limbic regions might influence the relationship among epilepsy and anxiety, future studies are required to further test this hypothesis.

Finally, we did explore whether the findings in NPY/PV would allow us to explain the absence of evolution to SE or SRS. Those animals that were injected with pilocarpine, but did not develop SE (NoSE) presented behavioral and neuroanatomical alterations as compared to control animals. Specifically, they showed altered density of NPY in various brain regions, and of PV in the piriform cortex, as compared to controls. This is very interesting, because as such animals did not develop SE, they consequently did not present exacerbated neuronal loss, classically evidenced in epileptic animals (Covolan and Mello, 2000). Moreover, animals in the Non-Anxious group presented characteristics of anxiety when re-tested, which would be correlated with the different nuances of expression of the neuromodulators that was found on these animals. The analysis of NoSE animals is usually not deeply explored in the literature, however, other studies also showed that NoSE animals were not always like control animals (De-Mello et al., 2005; Blanco et al., 2009), although less pronounced differences were also verified by others (Wolf et al., 2016). Additional studies are necessary to elucidate those findings. This points to the heterogeneity of the epileptogenic and behavioral response after an initial insult and should be considered as an important factor on pharmacological development.

## Conclusion

The discussion above highlights the complex involvement of neurotransmitter systems in different brain areas and of behavioral traits that hypothetically could suggest a strong link between anxiety and epilepsy. It has been suggested that anxiety disorders in a community population with epilepsy are most strongly predicted by factors independent of epilepsy-related variables (Mensah et al., 2007). Our own data in an epilepsy model corroborates that finding. The processes by which the association between anxiety and epilepsy arise might be a function of changes in neurotransmitter systems which are relevant in both conditions such as that involving NPY. By emphasizing the interrelationship between anxiety and epilepsy, this study may underline possible therapeutic targets to mitigate the potential negative effects resulting from this interaction.

## Data Availability

The raw data supporting the conclusions of this article will be made available by the authors, without undue reservation.
